# Book Review

**Published:** 1920-06

**Authors:** 


### Reviews of Books.

**Eugenics and Environment.** By Professor C. Lloyd Morgan, LL.D., D.Sc., F.R.S. Pp. 82. London: John Bale, Sons & Danielsson, Ltd. 1919. Price 1s. net.—In this readable little work Professor Lloyd Morgan has furnished us with a very clear *précis* of the relationship between Heredity and Race Culture. The train of thought is easy to follow from cover to cover, and is uninterrupted by statistical formulæ or technical phraseology. The author's remarks on the relative value of Nature and Nurture should give pause to those who are apt to regard Environment as capable of making good the deficits of Heredity. The vexed question of the inheritance of acquired qualities is ably dealt with in Chapter V. In this connection Professor Morgan illustrates the functions of the body cells and germ cells as analogous to the functions of the workers and the queen and drone bees in a hive; and he points out that the training of the "workers" in the brain of a mathematician, for example, may produce modifications of the tissues which we call acquired characters, but that, the workers being childless, these acquired characters cannot be transmitted in cellular filiation. We consider that this book should be in the hands of all persons who are engaged in eugenic or educational schemes.

**An Index of Prognosis and End Results of Treatment.** By various writers. Edited by A. Rendle Short, M.D., B.S., B.Sc., F.R.C.S., 2nd Edition, pp. xi., 770. Bristol: John Wright & Sons, Ltd. 1918. Price 30s. net.—This work, like the companion volumes on Diagnosis and Treatment, has already a high and assured position in the medical world, and we heartily welcome the present edition, which has undergone far-reaching revision, and has received important additions during the war. Thus Gun-shot Wounds, Gas Gangrene, Tetanus and Septic Peritonitis have new sections allotted to them while Tropical Diseases have been rewritten by Sir Leonard Rogers.

To those who have not yet made a study of the book we may point out the extreme interest of many of the articles, the immense number of facts accumulated as to prognosis, and the valuable comparisons as to the results of various lines of treatment. There can be no doubt as to the usefulness of the work to the practitioner. It has, however, a two-fold value. While it has been possible in a great number of diseases and injuries to show the prognosis and the results which may be expected from various lines of treatment, in others the work is a true Baconian history showing the existing gaps in our knowledge and the sections of medicine which offer a useful field for further study. Nothing more stimulating for future research can be imagined than the perusal of those chapters which define our present limited knowledge of certain disorders.

**Manual of Bacteriology.** By RICHARD MUIR, M.A., M.D., and JAMES RITCHIE, M.A., M.D. Seventh Edition. Pp. xxiv., 753. London: Oxford Medical Publications. 1919. Price, 16s. net.—Reiteration of the merits of a well-known text-book are superfluous, but something may be said about the changes found.

The seventh edition retains its compact form, and contains even a greater amount of reliable information than the previous edition. The form in which it is presented is difficult to assimilate. The caution of the authors so rigorously excludes untried theories, that it robs the book of interest by not showing the trend of recent thought. Cold water is thrown upon ion concentration research. All vouchsafed to the subject is a short paragraph in small print, in which the difficulties of Ph. estimations are exaggerated, and the ideas of ultrascientific refinement and impracticability suggested. Amino acids, too, are not even mentioned in the index, and there is no indication of their importance in media.

The lack of devices for guiding the attention prevents the correct impression being obtained by those commencing the study of bacteriology; for instance, students reading about streptococci (pp. 203–205) would probably miss the important factors in the differentiation of pathogenic from non-pathogenic varieties. The "organisms of war time" are treated at length, particularly the dysenteric and anærobic bacteria. Descriptions of trench fever and infective jaundice have been added. The section on influenza has not been changed much, and is disappointing.

Among other diseases the sections on actinomyces, pneumonia and poliomyelitis have been thoroughly revised, those on spirochætes and amœbæ have been enlarged.

The bibliography at the end has the great value of being an accurate and carefully selected list by recognised authorities, and the index is complete and accurate.

The high standard of work has been maintained by the publishers.

**Fitting-out and Administration of a Naval Hospital Ship.** By EDWARD SUTTON, Surgeon-Commander, R.N. Pp. vi., 110. Bristol: John Wright & Sons Ltd. 1918. Price 8s. net.—The book opens with a short account of the evolution of the hospital ship from its earliest inception down to the present-day type, together with useful suggestions as to future development. Next follows some account of the Geneva Convention, in so far as it touches naval warfare.

After these preliminaries an excellent account is given of the type of vessel most suitable for conversion into a hospital ship, and of the essential plant, staff, and accommodation which must be provided.

Details are given of the fitting out of the s.s. *Drina* for this purpose during the late war.

Hospital routine and administration are very fully dealt with, and many excellent methods have been devised by the author for simplifying the work, which might well be adopted in any civil hospital.

Numerous illustrative charts are given, which merit attention. Now that the war is over the book has lost much of its interest for the general medical reader.

For members of the Naval Medical Service, however, it remains a very valuable work, while those who are concerned with the administration of civil hospitals would do well to study it with a view to the adoption of some of the writer's orderly methods.

**The Nervous Heart.** By R. M. WILSON, Capt. R.A.M.C., and JOHN H. CARROLL, Major, M.C., U.S.A. Pp. vii., 136. London: Oxford Medical Publications. 1919. Price 6s. net.—In this little book the authors develop with enthusiasm a theory of causation of "irritable heart" as seen in soldiers. They ascribe this syndrome to the action of bacterial toxins on the vagus depressor system, rendering it hyper-excitable. The importance of the infective factor no one will deny, but it is no more independent of personal and other pre-disposing factors in this syndrome than it is in, say, tuberculosis. As to the vagus factor, the writers may or may not be right, it is impossible to judge from their book, because the evidence brought forward in support of their contention is of the slightest. The pathology of the autonomic nervous system is likely to become a fruitful field for speculation in the immediate future, and it is of the utmost importance that we should be content to explore this field by the Hippocratic plan—the methodical accumulation of solid facts.

